# Performance of Polydopamine Complex and Mechanisms in Wound Healing

**DOI:** 10.3390/ijms221910563

**Published:** 2021-09-29

**Authors:** Dantong Zheng, Chongxing Huang, Xuhao Zhu, Haohe Huang, Chenglong Xu

**Affiliations:** School of Light Industry & Food Engineering, Guangxi University, Daxue Road 100, Nanning 530000, China; zhengdt18@163.com (D.Z.); 2016301055@st.gxu.edu.cn (X.Z.); huanghaohe@st.gxu.edu.cn (H.H.); clxu@st.gxu.edu.cn (C.X.)

**Keywords:** polydopamine, photothermal, electrical stimulation, antibacterial, inflammation

## Abstract

Polydopamine (PDA) has been gradually applied in wound healing of various types in the last three years. Due to its rich phenol groups and unique structure, it can be combined with a variety of materials to form wound dressings that can be used for chronic infection, tissue repair in vivo and serious wound healing. PDA complex has excellent mechanical properties and self-healing properties, and it is a stable material that can be used for a long period of time. Unlike other dressings, PDA complexes can achieve both photothermal therapy and electro activity. In this paper, wound healing is divided into four stages: antibacterial, anti-inflammatory, cell adhesion and proliferation, and re-epithelialization. Photothermal therapy can improve the bacteriostatic rate and remove reactive oxygen species to inhibit inflammation. Electrical signals can stimulate cell proliferation and directional migration. With low reactive oxygen species (ROS) levels, inflammatory factors are down-regulated and growth factors are up-regulated, forming regular collagen fibers and accelerating wound healing. Finally, five potential development directions are proposed, including increasing drug loading capacity, optimization of drug delivery platforms, improvement of photothermal conversion efficiency, intelligent electroactive materials and combined 3D printing.

## 1. Introduction

Severe trauma can cause a great deal of bleeding [1,2]. Traditional and extensive use of hemostatic agents, including gauze [3], gelatin sponge [4] and bandages [5], can be used to seal the bleeding part of the wound surface by pressing or tightening the wound hard, thereby exerting a hemostatic effect. However, the hemostatic performance will greatly reduce without pressure. Therefore, traditional hemostatic dressing is not suitable for large and irregular surface wounds that are inconvenient to press, and uncontrollable surface bleeding from wounds of fragile visceral tissue [6,7,8,9]. This usually results in secondary injury or secondary pain for the patient [10,11,12,13]. So, it is necessary to design a non-oppressive tissue adhesive with strong adhesion that can quickly stop bleeding and relieve patients’ pain. Chronic wounds caused by diabetes, burns and other reasons have cell migration defects and prolonged inflammation during the recovery process, leading to increased fibroblast apoptosis, oxidative stress imbalance and excessively high levels of reactive oxygen species [14,15], and low function of collagen deposition [16,17]. The high level of reactive oxygen species will further promote the proliferation of bacteria, which cannot provide a good environment for the adhesion and proliferation of tissue cells. Therefore, it is necessary to design an antibacterial biomaterial that has the ability to correct or reverse the imbalance in diabetic wounds and has the inherent characteristics of inflammatory regulation and wound healing acceleration. PDA complex is an ideal wound dressing that meets the above requirements.

PDA complex refers to a type of wound dressing formed by compounding PDA with organic or metallic materials through sedimentation, grafting and cross-linking. PDA complex is mostly hydrogel, which can provide a moist and closed healing environment for wounds. PDA is made by polymerizing dopamine monomer. Dopamine is oxidized in alkaline solutions [18]. Recent studies have shown that PDA, with its photothermal conversion properties and strong adhesion, can be combined with many organic and inorganic molecules to prepare PDA complex dressings for different types of wound healing. PDA complex hydrogel and scaffold can mimic the matrix for cell attachment and growth. Additionally, due to their unique physical and chemical properties, they have been proven to generate a microenvironment with antibacterial, anti-inflammatory and electrical stimulation functions, which can guide cell behavior based on cell-material interactions. PDA can be combined with a variety of substances to change its mechanical properties, increase its electrical conductivity, improve and extend its antibacterial efficacy [19], and enhance its near infrared irradiation (NIR) function [20]. This article briefly outlines how PDA and different materials are combined, and compares their characteristics. The antibacterial mechanism of these materials and their regulation of the wound healing process is described. For the future development direction of PDA complex in wound healing, multiple levels of potential applications and improvement directions are proposed.

## 2. Construction Methods and Physicochemical Properties of PDA Complex

**Figure 1:**
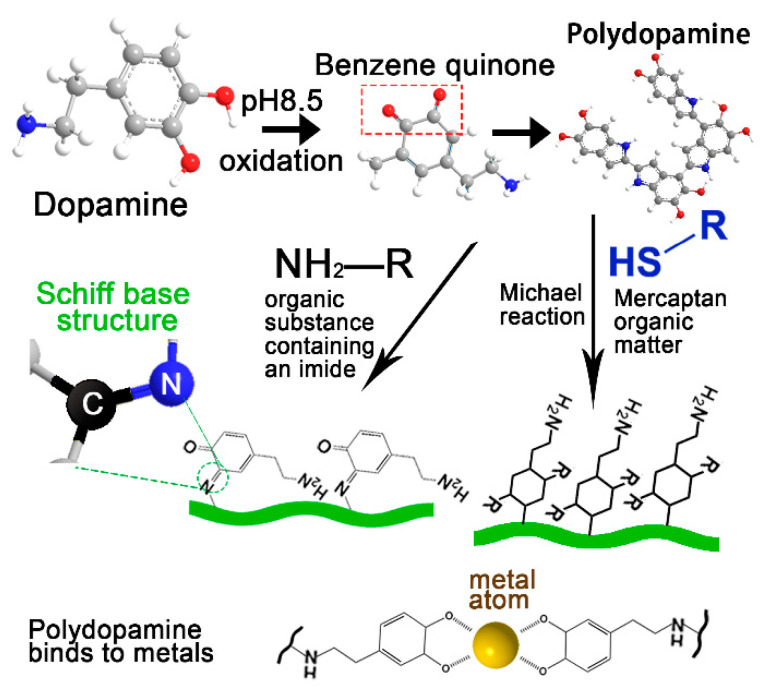
PDA complex fabrication.

PDA can bind a variety of organic materials through its rich catechol and amine groups and adhere to the organic surface and biological tissues, such as the heart, liver, lung, kidney and skin [21]. As can be seen from Figure 1, these interactions form nanoparticles, hydrogels, and electrospinning or surface coatings through physical and chemical cross-linking with covalent bonds [22] and non-covalent bonds [23]. Different construction methods and materials can make PDA complex have many properties which are beneficial to wound healing.

### 2.1. Mechanical Properties of PDA Complex

PDA is rich in phenol groups, so it shows strong adhesion to various tissues including human skin. This allows it to form a closed environment that promotes healing on the surface of wounds such as burns. However, as a medical dressing, PDA complex also needs to have mechanical strength similar to that of body tissue and skin, excellent ductility and compressive strength. Based on these mechanical properties, the advantages of stable covering, small displacement, long antibacterial effect and quick wound healing can be realized.

Non-covalently bonded hydrogels have better ductility and faster biodegradability compared to covalent hydrogels. The dopamine-talc hydrogel prepared by Jing et al. [24] elongated by more than 1000%. The axial compressive force of the gel increases with the increase in the PDA concentration [25]. However, simple non-covalent cross-linked materials often have insufficient mechanical strength. Therefore, covalent bonds are also required to form permanent crosslinks, resulting in higher crosslinking densities to produce better mechanical strength [26]. Ding et al. [27] added PDA to the gelatin hydrogel and reported that the weakest adhesion connection is the hydrogel interface in the uncoated gel material. In contrast, the weakest adhesion link in the coated PDA material is the cohesion of the hydrogel, which proves that the quinones of PDA forms a covalent bond with the lysine and arginine residues in gelatin. Similarly, the covalent bond between phenol in the alginate-PDA scaffold [28] contributes to the mechanical strength and structural integrity of the scaffold [29].

According to the reaction sequence, there are two types of covalent bonds between PDA and organic molecules. First, In the pH range of 8–8.5, phenolic hydroxyl is oxidized to benzoquinone. Through this in situ free radical polymerization, dopamine is easily deposited on almost any type or shape of the surface through oxidative auto-polymerization [30,31,32]. The covalent cross-linking network thus formed is relatively stable. Second, under weakly alkaline conditions, the adjacent quinones in dopamine undergo a strong Schiff base or Michael addition reaction with nucleophilic functional groups (such as amines and thiols) [33]. Benzoquinone can form a Schiff base structure with another dopamine molecule or an amino group in an organic substance and generate PDA. The Schiff base structure can be observed from the characteristic enhancement peak at 1653 cm^−1^ in the infrared spectrum. Therefore, the stent network is usually formed by the Schiff base reaction of PDA, rather than simply mixing. This structure can also improve the mechanical strength of the material by increasing the cross-linking density, but it is not as stable as the previous combination. This is because if the dynamic Schiff base network is interrupted over time, the support network may be disbanded [34].

### 2.2. Healing Properties of PDA Complex

**Figure 2:**
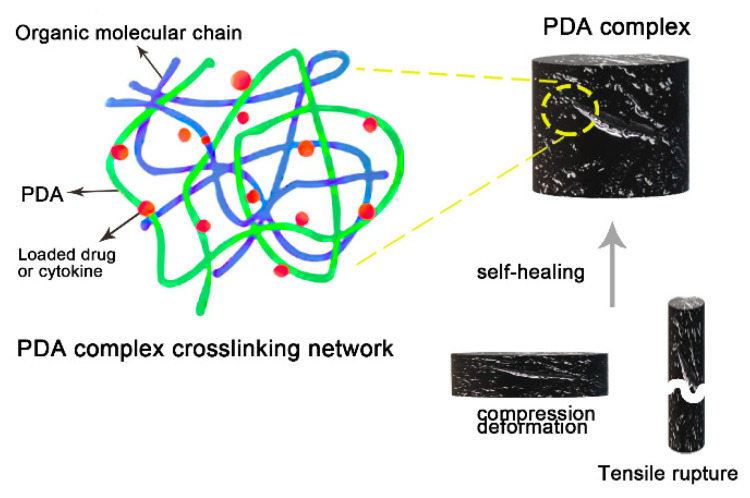
Self-healing of PDA complex hydrogel.

PDA complexes are mostly gel structures. The tensile strength and ductility of PDA give the gel excellent deformation properties. This means that gel structures can be deformed to absorb blood to stop bleeding, or deformed to release drugs. Traditional gels may be unable to recover their original shape or even damaged after excessive deformation, resulting in short service life. However, due to the rich functional groups of PDA itself and its unique cross-linked structure, the broken or compressed gel can restore its original shape (Figure 2). The self-healing properties of the gel indicate that the addition of PDA can maintain its structural integrity [35]. The self-healing gel structure containing PDA is divided into non-irritating type, pH-stimulating type and NIR-stimulating type. The non-irritating self-healing hydrogel is due to the redox properties of the composite material. For example, graphene oxide (GO) [36] can reduce the oxidized quinone to a hydroxyl group and re-polymerize to form a molecular chain. The pH-stimulated self-healing involves electrostatic interaction, and the hardness of the hydrogel also changes with the pH value [24,37]. The self-healing properties of PDA gel are mainly enhanced by NIR. Laser irradiation increases the activity of PDA and promotes the interaction between PDA and macromolecular chains through covalent/non-covalent bonds. The laser-induced heating increases the mobility of molecular chains in organic compounds [38,39]. The molecular chains on the crack surface diffuse and interact with adjacent PDA-NPs and repair the damage. The NIR stimulating PDA complex can also precisely control the shape, location and speed of gel healing by changing the radiation intensity of the light source, exposure time and irradiation location.

It is worth mentioning that once covalent bonds are destroyed after repeated healing cycles, it is more difficult to restore their original state [36,40]. So, compared with covalently bonded structures, non-covalent interactions show longer cycle self-healing ability because non-covalent interactions are reversible. For example, catechol groups on the PDA chain and the organic molecules containing hydroxyl groups can be connected through non-covalent bonds to form a recoverable network, effectively dispersing the PDA particles in the cross-linked network [40]. Or a benzene ring on PDA can be cross-linked with a positively charged amino group on an organic molecule via cationic-π interactions [41,42]. The supramolecular polymer obtained by this strategy can remain effective in multiple healing cycles without any loss of polymer properties or structural integrity [43].

### 2.3. Electrical Conductivity of PDA Complex

Cell adhesion and migration are regulated by functional proteins and ions, and its behavior may be regulated by appropriate electrical stimulation (ES) [44,45]. Changes in protein and ion levels caused by electrical signals may also change cell morphology [46]. This is because electric fields activate multiple cellular signaling pathways such as PI3K/PTEN, the membrane channel of KCNJ15/Kir4.2 and intracellular polyamines. These pathways are involved in the sensing of physiological electric fields, directional cell migration, and possibly other cellular responses [47]. Electrical stimulation can be divided into exogenous ES and endogenous ES. The application of exogenous ES in the healing of a skin wound may require the use of large extra corporeal electrical devices, causing inconvenience to patients. In a skin wound that destroys the epithelial barrier, the trans epithelial potential at the wound that completely penetrates the epidermis is zero, establishing a potential gradient or endogenous current from the undamaged epidermis to the wound [48]. Recent work has found that wound-induced current can stimulate tissue growth; this phenomenon is called the electrical axis or electrotaxis [49]. Endogenous ES can guide the migration and proliferation of cells to the wound along the gradient, regulate cell proliferation, aspect ratio and gene expression, and promote vascular differentiation and tissue maturation on the wound surface [50] until wound healing and rebuild the original skin battery. Therefore, providing conductive pathways to enhance endogenous ES is a more convenient way to promote wound healing. This endogenous wound current decreases with the increase in electrical resistance during wound healing, so electrical resistance can be used to measure wound healing [51].

Conductive materials may make endogenous/exogenous ES more efficient, directing/aligning cell migration to the wound, ultimately accelerating wound regeneration [52]. When PDA is complexed with conductive materials, ES will be further expanded. Most traditional electroactive materials lack cell affinity and have poor processing performance, such as polyaniline, PPy, and PEDOT. In addition, nano-conductive materials are difficult to disperse in the matrix, and the conductivity of the composite dressing is limited by the dispersibility of these conductive materials. Improving the dispersion and cell compatibility of conductive nanomaterials in a biocompatible matrix has become a key point in the manufacture of electrically responsive wound dressings [53]. PDA can not only overcome the limitations of the mechanical properties and processing performance of electroactive materials, but also make the materials more conductive than ordinary reducing agents. First, the polymerization of PDA on the surface of the material protects it from any further attack by alkaline solutions. Additionally, the catechol on the PDA layer is not only an active site that can induce the deposition of other substances, but also prevents the agglomeration of cellulose and other substances, uniformly distributes organic matter, provides suitable conductive pathways [54] and constructs a cellular communication network. Electrical signal stimulation generated by the material can further regulate cell differentiation. Additionally, the PDA-modified nanosurface can also guide the directional migration of cells.

In addition to electromechanically active materials, metals also conduct electricity. Metal-based materials such as copper, iron, silver, zinc, and titanium have been used as conductive materials for wound healing and are usually prepared in various nanometer forms for such applications [55]. For example, the covalent chemical grafting of aromatic organometallic compound EDC-NHS on the PDA coating significantly improves the conductivity of the stent, and the product can be applied to devices such as biosensors [56]. On the nanoscale, these metal-based materials can be easily chemically or physically modified due to their large surface area, and exhibit good biocompatibility and significant electrical conductivity [57]. Except chemical grafting, the catechol group on the PDA chain can usually also change the biological function of the metal surface through strong coordination bonds [58].

## 3. The Mechanism of PDA Complex for Promoting Wound Healing

The PDA coating can convert a hydrophobic surface to a hydrophilic surface [59], providing a moist and airtight environment for wound healing [60], which can enhance the affinity of the material to cells/tissues and promote their adhesion to them. This is because PDA has an excellent interfacial binding affinity to the nucleophilic moieties (-NH2 and -SH) that usually exist on the surface of biological tissues such as the liver, heart, spleen, lung, kidney, and tail [61,62,63]. It has been reported that the adhesion strength of a PDA complex to pigskin can be as high as 90 kPa [35]. Combining the adhesive property with the superior ductility, mechanical property and self-healing property of PDA complex enables PDA complex to form a long-term and stable healing environment on the wound surface. This avoids the secondary tearing of the wound caused by the need for repeated replacement of traditional dressings [64]. 

**Figure 3:**
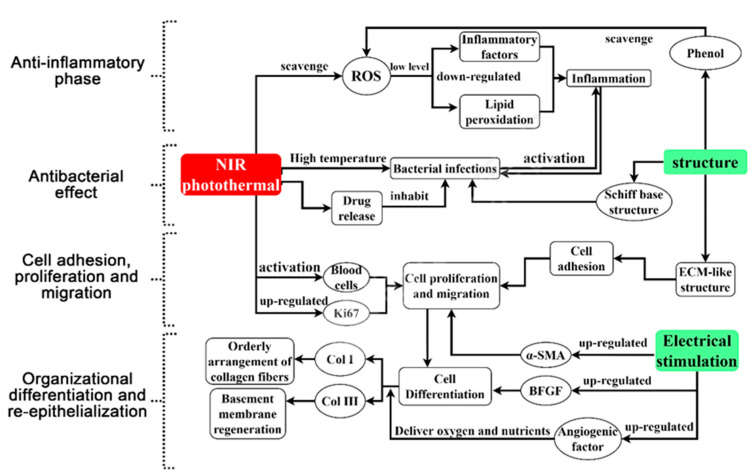
Mechanisms of PDA complex in wound healing.

Wound healing is a continuous and overlapping process, which can be divided into four stages: antibacterial, anti-inflammatory, cell adhesion and proliferation, tissue differentiation and re-epithelialization. Mechanisms of the PDA complex on the different stages of the healing process also influence each other (Figure 3). PDA complex promotes wound healing in three main ways. First of all, the photothermal properties of PDA complex have bacteriostatic, anti-inflammatory and growth factor regulation functions, which suggests that the photothermal effect acts on the whole process of wound healing. Second, PDA complex not only has strong tissue adhesion, but also provides a variety of cell adhesion sites. In addition, PDA cross-linked electroactive materials not only improve the processing and mechanical properties of traditional electroactive materials, but also create an endogenous bioelectrical pathway leading to the wound, guide cell migration and orientation, and increase the deposition and arrangement of collagen. The effects of PDA complex on wound healing are not limited to these two areas, but extend throughout the healing process. Most PDA wound dressings can close full-thickness defect wounds within about 15 days, but the degree of re-epithelialization may vary depending on the composition. At present, the best effect is the quaternary ammonium chitosan (QCS)/PDA material, which has the greatest re-epithelialization effect, and the process can be completed within 10 days [25].

### 3.1. Antibacterial Effect

Clinically, bacterial infection and biofilm formation often occur on living tissues and/or various medical devices, accompanying inflammation responses. Severely, mature biofilms release planktonic bacteria that trigger new infections and further activate the immune system to produce inflammatory responses. The persistent inflammatory response would eventually lead to cell death and tissue necrosis, trigger surrounding soft tissue inflammation or damage. Previous studies have shown that excessive inflammatory response is also one of the main reasons for delaying wound healing, resulting in hypertrophic cicatrix formation [65]. On the other hand, an excess of bacteria cannot provide a suitable environment for cell proliferation. Therefore, the primary task of preventing wound infection is to inhibit bacteria. 

**Figure 4:**
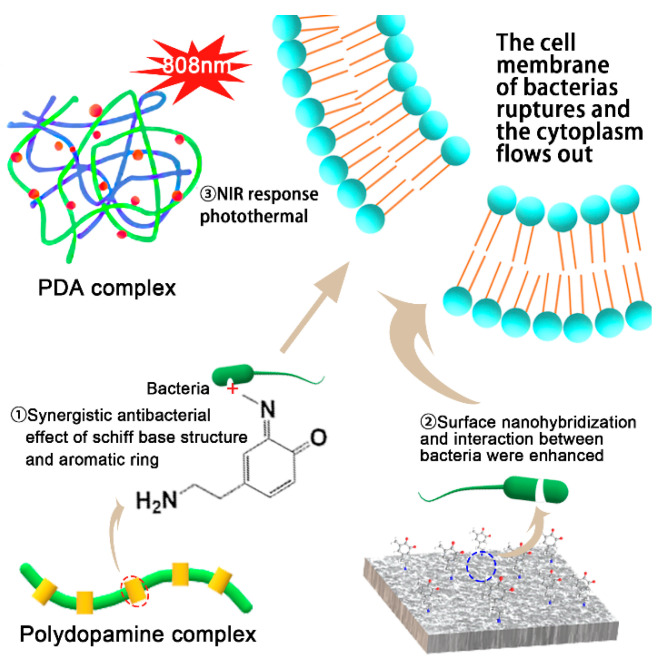
Antibacterial mechanisms of PDA complex.

The PDA coating alone lacks efficient and stable antimicrobial activity. Therefore, it is often necessary to complex with bactericidal substances (Figure 4) [66]. From the structure of PDA complex, the Schiff base structure of PDA has a synergistic antibacterial effect with the aromatic ring. PDA can also enhance the effect of antibacterial by the interaction between nano-hybridization and bacteria. The main source of antibacterial power of PDA and its NIR response to photothermal effect. During the heating process, the temperature difference and antibacterial efficacy increase with the increase in PDA content. After PDA coating, the absorbance at 808 nm increased significantly. This may be due to the introduction of a lone electron pair (-OH) in the PDA, increasing the molecular conjugation system through resonance, thereby increasing the range of electronic activity [33].

By summarizing the bacteriostatic rate data of some PDA complex, it was found that the inhibition rate of the complex was higher under NIR radiation and the complex was generally more effective against Gram-negative bacteria (Table 1). This is because the high temperature produced by NIR can accelerate the death of bacteria. However, *Staphylococcus aureus* (*S. aureus*) and other Gram-positive bacteria are generally more heat-resistant than Gram-negative bacteria such as *Escherichia coli* (*E. coli*). The former has a thick peptidoglycan layer (20–80 nm) composed of amino acids, surface proteins, teichoic acid and lipids. Gram-negative bacteria lack these structures. Lack of outer membrane promotes the penetration of the surrounding environment through the peptidoglycan layer [67].

There is a difference of approximately 20 °C in the NIR temperature rise of other compatible materials. However, the temperature difference of the composite material containing PDA and metal cations is 40 °C. When the temperature exceeds 50 °C, the protein will irreversibly denature, leading to rapid bacterial death. This means that when PDA is complexed with antibacterial metal cations, its photothermal and antibacterial effect are enhanced. Pure copper ions only gradually inhibit bacteria. However, laser irradiation quickly heats metal cations and accelerates the inhibition of bacterial growth. However, after the laser irradiation is terminated, the antibacterial effect is terminated. The combined effect of copper ions and laser irradiation provides rapid and long-lasting antibacterial effects [70]. The intensity and duration of irradiation in different studies are slightly different. Although the addition of PDA prevented the aggregation of nano-metal particles [79], which led to the decrease in the antibacterial effect of the metal itself [80], the overall antibacterial effect of the composite was enhanced due to the photothermal effect. Moreover, PDA coverage can also prevent the contact between AuNPs and cell and reduce the cell toxicity of AuNPs [25].

On the other hand, metal cations also inhibit bacterial growth by inhibiting the formation of biofilms, changing membrane permeability, producing ROS that interferes with RNA and DNA replication [81] and inducing genotoxicity [82]. It is worth noting that this strategy of suppressing bacterial growth by producing ROS can only be used in the early antibacterial process of wound healing. This is because the pH of the severely infected site can be 5.0–5.5, and there is significant hydroxyl generation at this pH value [83]. PDA will also accelerate the production of antibacterial active oxygen after the laser is irradiated and heated [84]. Although it can quickly kill bacteria in the early stage, but this will affect the inflammatory response in the middle of the wound and tissue maturation in the later stage. This strategy is also not suitable for diabetic ulcers, because excessive levels of inflammatory cytokines and ROS and defective cell function can impair diabetic wound healing [56].

In addition to compound with antibacterial substances, PDA can also produce targeted and sustained antibacterial effects by loading and releasing antibacterial drugs. PDA has a large number of catechol groups, benzene rings, delocalized π electrons and C=C double bonds. Therefore, the PDA coating will react specifically with biomolecules containing sulfhydryl, amine and amino groups [85]. Existing studies have found that drug molecules can be loaded into PDA nanoparticles through π-π stacking, electrostatic attraction, and hydrogen bonding [80] and achieve sustained drug release, response release, or targeted release by adding specific ligands. The sustained drug release characteristics of PDA nanoparticle-coated hydrogels can be attributed to the high binding ability of PDA with organic matter through Michael addition [86] or Schiff base reaction [87] or the π-stacking reaction between small drug molecules and PDA [88]. These interactions will inhibit the pulsed drug release and prolong the drug release time.

The responsiveness of drug release is also affected by the concentration gradient, pH responsiveness and NIR [89]. Previous studies have involved loading curcumin into MPDA via π-π stacking and hydrogen bonding. Under NIR irradiation, π-π stacking and hydrogen bonding were affected, which resulted in release of curcumin from MPDA. The PDA hydrogel with large pores inside, after NIR, the macroporous structure collapses, which will also cause large volume shrinkage [35] and drug release [36]. The pH-dependent behavior of drug release can be explained by various electrostatic interactions between PDA and drug. The amino group of PDA deprotonates at high pH and generates negative charges. However, at low pH, PDA is protonated [36].

### 3.2. Anti-Inflammatory Effect

PDA complex can regulate inflammation and growth factors, regulate immune response, increase fibroblast activity, protect fibroblasts from apoptosis, and weaken the inflammatory response at the wound site. Inflammation affects the speed of wound healing and scar tissue formation. The specific performance is that PDA complex can help down-regulate pro-inflammatory cytokines, regulate the immune response, up-regulate angiogenic factors such as CD31 and VEGF [90] and promote wound vascular differentiation and tissue maturation. CD31 is a transmembrane protein expressed in early angiogenesis. It is used to confirm the presence of endothelial cell tissue and assess angiogenesis. VEGF affects the migration, proliferation and angiogenesis of vascular endothelial cells [91,92,93].

The large amount of ROS produced by neutrophils at the wound site may destroy biological macromolecules. The PDA catechols have antioxidant effects [94]. Phenol groups can capture electrons and scavenge ROS. Excessive ROS production and lipid peroxidation can hinder the healing of chronically infected wounds such as diabetes [95]. The phenol group in PDA is converted to quinones, and the free radical redox equilibrium is established [50], and this activity varies with the content of dopamine in the complex [96]. In contrast, the phenoxy group produced by the conversion of catechol to benzoquinone can be stabilized by electron delocalization. The reduction of ROS levels in macrophages can reduce the expression of pro-inflammatory factors and promote tissue regeneration. Pro-inflammatory cytokines include IL-6, IL-1β, TNF-α and CD86. IL-6 is closely related to the severity of inflammation caused by bacteria and is a sensitive indicator for clinical diagnosis of bacterial infections [97]. IL-6 promotes the release of somatostatin and inhibits the release of growth hormone [98]. PDA material significantly down-regulates many pro-inflammatory factors obtained from wounds [54].

For chronic wounds like diabetes, the degree of oxidative stress in the wound can also be assessed by measuring lipid peroxidation and antioxidant enzyme activity [99]. Superoxide dismutase (SOD) is an enzyme in the antioxidant defense system of skin tissue. In diabetic wounds covered by PDA dressings, SOD is up-regulated, which means that oxidative stress is reduced. Matrix metalloproteinases (MMP) are zinc-dependent endopeptidases [100]. In diabetic animals, high levels of MMP-2 and MMP-9 inhibit multi-factor homeostasis and re-epithelialization in the extracellular matrix [101], tissue fluid [102] and diabetic foot ulcer tissue [103]. Excessive ROS can abnormally up-regulate MMP in diabetes [104]. After PDA clears ROS, it down-regulates MMP-2 and MMP-9, reduces excessive tissue proteolysis, and promotes diabetic wound healing.

### 3.3. Cell Adhesion, Proliferation and Migration

Blood cells, keratinocytes, endothelial cells, fibroblasts, inflammatory cells and other cell types are involved in wound healing [105]. PDA dressings promote cell adhesion, proliferation and migration in four ways. Among them, it mainly promotes wound healing by precisely regulating various cell responses and cytokines in the process of homeostasis, inflammation, granulation formation and remodeling. The functional groups on PDA-NPs (including catechol and quinone) promote cell adhesion and proliferation, and form covalent/non-covalent interactions with adjacent tissue surfaces. In addition to the electrical stimulation mentioned above, PDA complex promotes cell adhesion, proliferation, and directional migration through three other pathways.

#### 3.3.1. Simulation of the Extracellular Matrix (ECM)

The unique nanostructure and abundant sites of PDA material can mimic the adhesion of ECM and become binding sites for various proteins and differentiation sites for various cells. The nano-crosslinked network in the scaffold is similar to the collagen fibers in ECM. This is beneficial for the distribution, migration, proliferation of cells and reconstruction of damaged tissues. Therefore, PDA can be combined with other materials to form scaffolds with low cytotoxicity and excellent biodegradability. Among them, the degree of cell damage and death under the dressing can be quantitatively determined by the lactate dehydrogenase (LDH) release method [34]. Fluorescence-labeled bovine serum albumin (FITC-BSA) can measure the amount of protein adsorbed on the film and evaluate its biocompatibility [60]. Previous studies have reported that PDA has negligible toxicity to 3T3 fibroblasts and human umbilical vein endothelial cells (HUVEC) [106]. Due to the interaction sites provided by dopamine, EF1 human fibroblast cell cultures and PDA hydrogels show good biocompatibility [24].

The porous ECM-like network modified scaffold formed by the composite of PDA is a microstructure with enhanced porosity, specific surface area and oxygen permeability. They also protect wounds from external contamination and further promote cell adhesion and proliferation. The porous structure may be penetrated and filled with the extracellular matrix (including fibrin, collagen molecules, and lysyl oxidase) [107]. Lysyl oxidase and collagen itself can be cross-linked to obtain long-term strength and stability [108].

PDA nanoparticles can promote cell protrusions, such as flakes and filamentous feet, and promote cell proliferation through expansion [109]. The cells adhered to the PDA scaffold formed superior α-smooth muscle actin (α-SMA) protein bundles and provided greater adhesion [34]. Certain non-structural proteins in ECM also play an important role in cell adhesion and proliferation. Abundant active groups in PDA mimic these proteins and provide adhesion sites. Additionally, the adhesion strength increases with the increase in PDA content [40,110].

#### 3.3.2. Activated Blood Cells

Some PDA complexes can fix platelet-rich plasma to achieve the functions of coagulation and activation of platelets. The macroporosity in the scaffold or cross-linked gel supports and improves blood cell adhesion and enhances coagulation [111]. Various growth factors in the plasma combined with NIR stimulation can regulate the formation of blood vessels and the proliferation and differentiation of fibroblasts. Platelet-rich plasma (PRP) is a source of various growth factors, such as PDGF, IGF-1, VEGF, FGF-2 and so on. It stimulates the formation of new blood vessels and various fibroblast activities [112]. Vascular endothelial growth factor (VEGF) is the main angiogenesis inducer. Platelet-derived growth factor (PDGF) expands blood vessels and forms mature blood vessels, which has a strong chemotactic effect on fibroblasts and smooth muscle cells. The adhesion of platelets to the gel increased with the increase in PDA content.

In addition, PDA gel causes their morphology to change from a disc to an irregular shape. The status and number of platelets determine the amount of bleeding and clotting time [75]. The gel absorbs a large amount of blood at the bleeding site during the expansion of the shape, prevents the adhesion between the tissue and the hemostatic device, and protects the tissue from the destruction of blood flow. In contrast, the interconnected large porous structure inside the compressed gel can restore its original shape and specific surface area, strengthen the adhesion of blood cells and platelets, and concentrate and activate the hemostatic agent [25].

#### 3.3.3. NIR Irradiation Promotes Cell Proliferation

NIR-mediated phototherapeutic possesses the advantages of higher tissue penetration depth, great target selectivity, benign tissue compatibility, and avoidance of drug- resistant bacteria [113]. Chen et al. [105] showed that the PDA complex can effectively promote cell proliferation and reduce the apoptosis rate after NIR irradiation. When the NIR lamp is turned on, PDA-NPs generate heat locally through the photothermal effect. At temperatures above the lower critical solution temperature (LCST), hydrogel changes from a swollen hydrophilic state to a collapsed hydrophobic state and releases cells. On the other hand, on the 10th day after NIR irradiation, the stent with PDA significantly up-regulated Ki67 and Bcl-2. Ki67 is a nuclear antigen expressed in proliferating cells, which further indicate that the heat generated by infrared radiation helps the proliferation of cells, thereby further promoting wound healing.

### 3.4. Organizational Differentiation and Re-Epithelialization

Wound healing is a complex and dynamic process. This process is continuous and overlapping, including cell adhesion, cell proliferation, fibroblast transplantation, connective tissue synthesis, collagen deposition, wound re-epithelialization, and the formation of skin appendages. Tissue differentiation accelerates after controlling wound infection, because the down-regulation of inflammatory factors is often associated with the up-regulation of angiogenesis factors. Re-epithelialization is mainly driven by the migration and proliferation of keratinocytes. Keratinocytes are hormone receptors and can synthesize hormones that initiate wound healing [114]. Hormone production further stimulates wound closure. Most PDA complexes for chronic wounds are about 14 days, and the wound closure rate is about 98% (Table 2). They cover them with relatively thick new epidermis and more dermal papillae and hair follicles, and form a mature epithelial structure.

The photothermal effects, carrier function and electrical stimulation mentioned above also affect tissue differentiation and re-epithelialization. Photothermal therapy of PDA complex is safer than previous strategies of promoting wound healing by heating up, because it doesn’t change the cold environment of the subcutaneous tissue [115]. Such as catalyzing water/H_2_O_2_ at the site of bacterial infection [131,132] and delivering oxygen to the subcutaneous tissue [133] in an attempt to overcome hyperthermia. Some studies have injected growth factors directly into the injured full-thickness tissue to promote healing or injected plasmids or viruses with growth factor genes to up-regulated growth factors [134,135]. However, this method is influenced by tissue density, and the amount of reagents actually injected is limited, so the injection is easy to escape, which affects the therapeutic effect. PDA can be used as a suture carrier to deliver drugs or growth factors to deep wounded external or internal tissues [136,137].

**Figure 5:**
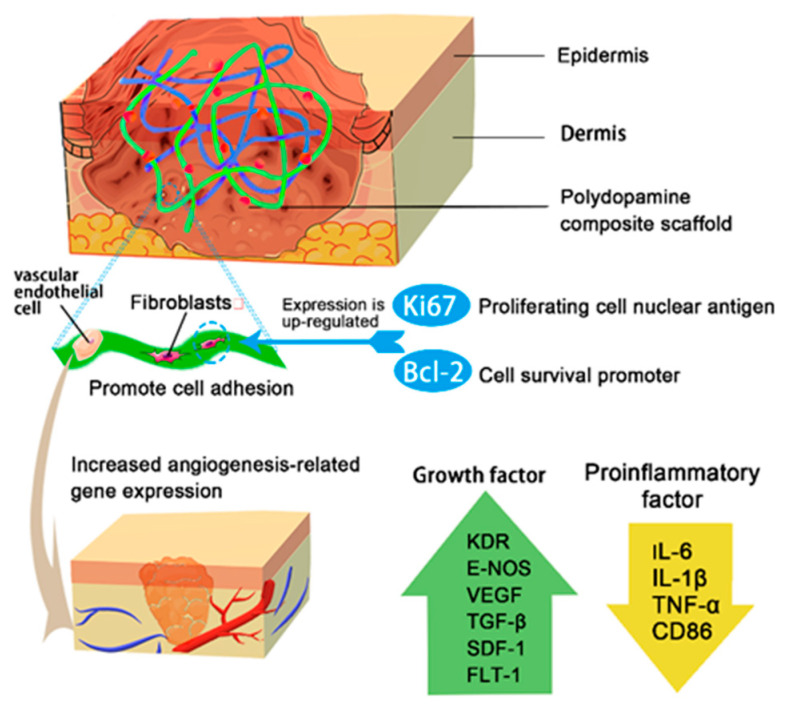
Regulation of wound tissue differentiation by PDA dressing.

Endogenous electrical signal stimulation generated by the conductive pathway provided by the PDA complex can up-regulate certain genes, build a cellular communication network, and promote the expression of a variety of proteins and cytokines that promote re-epithelialization. For example, smooth muscle actin (α-SMA) plays a major role in wound healing by promoting the proliferation and differentiation of fibroblasts. Collagen type III (Col III) plays a major role in granulation tissue reorganization and basement membrane regeneration. Furthermore, VEGF is linked to vascular endothelial cell migration and angiogenesis. The PDA scaffold accelerates wound closure and promotes wound healing by up-regulating α-actin, ColIII, platelet-derived growth factor (PDGF) [71] and VEGF (Figure 5) [52].

It can be seen that photothermal therapy, carrier transport and electrical stimulation mainly regulate multiple growth factors to achieve tissue differentiation and re-epithelialization. The most involved are angiogenic factors. Angiogenesis, the first step of tissue differentiation, is a key factor in wound healing. QRT-PCR analysis showed that PDA complex up-regulated genes related to angiogenesis, such as KDR, E-NOS, VEGF, TGF-β, SDF-1, and FLT-1. The cytokine TGF-β promotes the proliferation of fibroblasts and differentiates them into myofibroblasts. It also involves in matrix remodeling and angiogenesis. SDF-1 is an essential medium for wound healing. It recruits mesenchymal cells to the wound site and promotes IL-10 mediated angiogenesis [65,88] CD31 and α-SMA immunofluorescence labeling can be utilized to observe the effect of composite scaffolds on wound neovascularization. About 7 days after wound healing, there were significantly more CD31 cells in wounds treated with PDA materials than wounds treated with other materials. This suggests that the PDA complex promotes the formation of new blood vessels on the wound surface [115]. When the wound is restored and reconstructed, some small blood vessels degenerate and CD31 is down-regulated [138].

**Figure 6:**
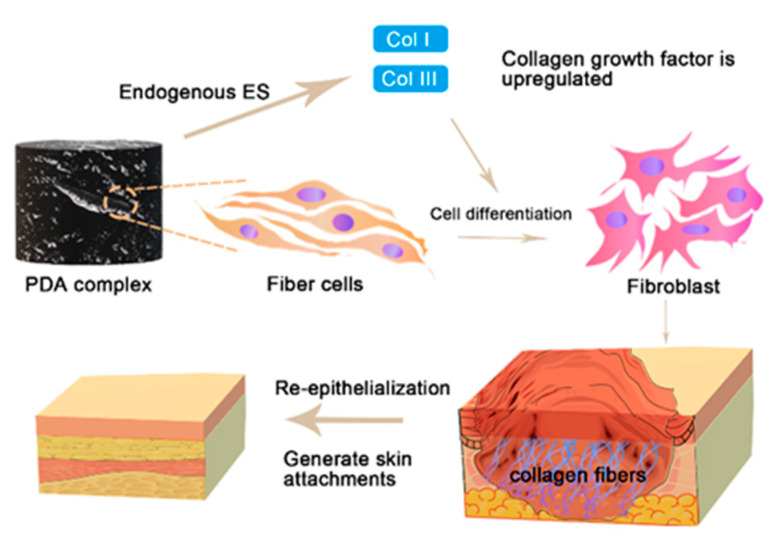
Re-epithelialization of the skin.

The newly formed blood vessels provide nutrients and oxygen to the wound bed, where they initiate collagen deposition and epidermal regeneration [139]. The degree of maturation of epithelial structures can be distinguished in a variety of ways. The wound surface, basement membrane thickness and cell arrangement both reflect the quality of the wound healing. H&E, Masson trichrome staining and MT staining [41,116,123] can show the cell arrangement and fiber content of wound at each stage. These techniques are associated with PCR to evaluate the expression levels of basic fibroblast growth factor (bFGF), type I collagen (Col I), Col III, TNF-α and other genes, and to evaluate re-epithelialization in wounds And collagen deposition. Collagen content treated with PDA complex usually increases within the first 10 days after the wound matures is stable, but it is also slightly different due to different types of collagen (Figure 6) [71]. PDA complex up-regulates Col I during the entire wound healing process, up-regulates Col III in the early and mid-healing stages, and down-regulates Col III after healing [69].

Finally, the wound treated with PDA complex showed an orderly arrangement of collagen fibers, abundant collagen bundles, a narrow gap of wound granulation tissue, and thick epithelium. Masson staining shows that the mature skin tissue induced by PDA complex is similar to natural skin tissue. It can obviously improve the deposition and arrangement of collagen [107] and decrease the formation of scars. As the wound heals and the new tissue are formed, the cell density gradually increases until the epidermal thickness and cell density are close to normal skin.

## 4. Conclusions and Prospect

PDA complex provides a kind of healing material that can meet the needs of rapid hemostasis, no compression, strong adhesion, high antibacterial rate and inflammation inhibition for wound healing. Excellent adhesion, mechanical properties and ductility provide a close-fitting healing environment. Combined with the self-healing properties of the PDA complex, the dressing lifespan is greatly extended. As PDA can provide a conductive pathway, when combined with electroactive materials, it can produce signals that regulate cell proliferation and directional migration. From different stages of wound healing, PDA complexes can enhance antibacterial activity through their own structure and photothermal effect, and can also achieve the purpose of targeted and continuous antibacterial activity by loading drugs. By simulating ECM and activating blood cells, NIR stimulation can further promote cell adhesion and proliferation. The photothermal effect and rich phenol groups of PDA complex can remove ROS to achieve the anti-inflammatory purpose. Finally, the wound was rapidly re-epithelialized by adjusting various growth factors.

The future development direction of PDA dressing can be considered from the following aspects. First, the mechanism of PDA polymerization has not yet been completely elucidated. Under alkaline polymerization conditions, PDA polymerization cannot ensure the loading or activity of small molecules. Preceding reports mostly used PDA as a wall material to encapsulate drugs, or load drugs on another material, which limited the loading capacity of drugs. In addition to the reported porous structure of mesoporous PDA that can increase the loading capacity [36,140], to better solve this problem, it may be possible to try to covalently bind the drug to the dopamine monomer and then polymerize it. Second, the drug delivery platform needs to be optimized. New drug delivery platforms should simultaneously realize sustained release, stimulated release, targeted release, and sequential release to assure better therapeutic effects [53]. PDA dressings can only achieve sustained release, targeted release, and stimulated release at present. In the future, the sequential release will achieve engineered and functionalized drug-controlled release.

Third, the efficiency of light-to-heat conversion needs to be improved. PDA has NIR response photothermal characteristics, and provides photothermal antibacterial ability with increasing temperature. The temperature difference of NIR treatment increases with the increase in PDA content by 20 °C, while for PDA complexed with metal cations, the temperature difference is 40 °C. However, for wound dressings used to bond tissues and organs in the body, metal materials are not easily degraded. Therefore, in the future, improving the photothermal conversion efficiency and photosensitivity of PDA and non-metallic materials is also a major direction for future research on PDA. This may not only speed up wound healing in internal tissues, but also may be used to treat tumor cells.

Fourth, the establishment of dual-function smart electroactive materials. In recent years, advances in real-time monitoring of wound conditions such as temperature, pH, etc., will allow more timely management of wound bed infections [141]. Furthermore, compared to some currently used materials, such as polyaniline, polypyrrole and polyethylene phthalate, the electrical conductivity may decrease over time due to the reduction in dopants in their physiological environment or loss [142]. The PDA can maintain a relatively constant conductivity in the physiological solution, which means that it can be combined with the method of measuring the transcutaneous resistance or impedance of the wound every day using electrodes to effectively measure the wound state [143,144], because resistance and impedance both increase with the progress of wound healing. However, the difference between the electrical resistance (impedance) of the conductive biomaterial and the electrical resistance (impedance) of the skin tissue must be carefully considered. This makes the PDA composite material a dual-functional active dressing that can simultaneously promote cell activities related to wound healing and monitor the healing process. It may help to achieve individualized treatment at different stages of wound regeneration and repair, and accelerate these processes.

Fifth, the combination with 3D printing. Compared with diabetic and chronically infected wounds, full-thickness skin defects caused by severe trauma and extensive burns are more complicated and take more time to heal [145]. Since PDA gel can maintain a fixed shape after preparation, it may not be suitable for wounds in large-area, total-loss skin models. Therefore, another future research goal is to combine PDA gel with 3D printing technology [146] to prepare dressings that fully fit large-area, irregular wounds.

## Figures and Tables

**Table 1:** NIR heating temperatures and bacteriostatic rates of various materials containing PDA.

Material	ΔTs/°C	NIR Antibacterial Rate	Anti-Bacterial Rate	Reference
Dibenzaldehyde-grafted poly (ethylene glycol) (PEGDA), lauric acid-terminated chitosan (Chi-LA), and Cur-loaded mesoporous PDA nanoparticles (PDA@Cur)	24.8	*E. coli*, 97.8%; *S. aureus*, 94.2%	*E. coli*, 37.1%; *S. aureus*, 20.4%	[68]
PDA@gold nanoparticles-hydroxyapatite PBS liquid	18	*E. coli*, 96.8; *S. aureus*, 95.2%	*E. coli*, 34.6%; *S. aureus*, 13.7%	[69]
Quaternized chitosan/PDA	27.2	*S. aureus*, 100%; *E. coli*, 100%	*S. aureus*, 100%; *E. coli*, 70%	[25]
TiO2 nanorods-PDA-Ferrocene	38	MRSA ^1^, ≥99%; *E. coli*, >99%	-	[56]
PDA/Cu-CS	43	MRSA, 97.64%; *E. coli*, 96.27%	MRSA, 13.89%, *E. coli*, 48.82%	[70]
Poly(L-lactic acid)-poly(citrate siloxane)-curcumin@PDA hybrid nanofibrous scaffold (denoted as PPCP matrix)	21	*E. coli*, 93.3 ± 1.2%; *S. aureus*, 97.7 ± 0.7%	-	[71]
Ag-pDA/BC (rGO)	-	*E. coli*, >84%	-	[72]
Deoxyribonuclease (DNase)-carbon monoxide (CO)@mesoporous PDA nanoparticles (MPDA NPs)	23	MRSA, 92%	-	[65]
bacterial cellulose/PDA/polyacrylamide hydrogels	-	-	*S. aureus*, 100%	[73]
MOF-PDA	31	*S. aureus*, 99.62%; *E. coli*, 99.97%	0	[74]
Gelatin/dopamine cryogels	24.2	*S. aureus*, 100%; *E. coli*, 100%	*S. aureus*, 70%; *E. coli*, 68%	[75]
DAP-GCS-PDA@GNRs	35	MRSA, 100%	-	[76]
MoS2@PDA-Ag	43	*S. aureus*, 99.99%	-	[55]
Carbon quantum dot (CQD)-decorated ZnO (C/ZnO) composites were chosen as the functional NPs	30	*S. aureus*, 99.9996%%; *E. coli*, 99.9998%	*S. aureus*, 70.8%; *E. coli*, 60.2%	[77]
GT-DA/CS/CNT gelatin-grafted-dopamine (GT-DA) and PDA-coated carbon nanotubes (CNT-PDA)	26.7	*S. aureus*, 100%; *E. coli*, 100%	*S. aureus*, 5.9%; *E. coli*, 2.1%	[78]

^1^ MRSA, Methicillin-resistant Staphylococcus aureus.

**Table 2:** Wound healing time and healing status of various PDA compound dressings.

Materials	Healing Time/d	Healing Condition	Reference
Bioactive glass/PDA-modified electrospun scaffolds	15	The wound was almost completely healed and the remaining wound area was 0.98%. There were more dermal papillae and hair follicles on the wound surface and the epithelial structure was mature.	[115]
PDA-NPs/PNIPAM gel	15	The wound was closed, mature skin tissue was regenerated, and collagen fibers and hair follicles were arranged.	[35]
QCS/PDA	10	The wound healed completely.	[25]
PDA-reduced graphene oxide (pGO)-incorporated chitosan (CS) and silk fibroin	21	Blood stagnation and wound closure were observed.	[94]
PDA-coated *Antheraea pernyi*	14	The wound was completely healed and new skin and hair formed.	[60]
Poly(glycerol-ethylenimine),Ti3C2TxMXene@PDA (MXene@PDA) nanosheets and oxidized hyaluronic acid (HCHO)	14	The wound healing rate was 96.31%.	[34]
PDA (PDA) coating on hydroxyapatite (HAp) incorporated with gold nanoparticles (Au-HAp)	10	The wound healed.	[69]
Poly(3,4-ethylenedioxythiophene)-PDA-silk microfibers	15	The wound healed.	[50]
PDA functionalized bioactive glass nanoparticles (BGN@PDA)-F127-ε-Poly-l-lysine hydrogel	14	The wound was largely healed and abundant granulation tissue was visible.	[110]
PDA@Ag NPs), polyaniline-polyvinyl alcohol	20	The wound completely healed.	[40]
PDA/collagen sponge scaffolds	21	There was a full-thickness skin defect on the wound surface. After 21 d, the full thickness skin of each group survived, the wound was closed, and there was no obvious gap between the skin and regenerated tissue.	[116]
pDA-epsilon PL/NFDS	32	The average burn wound healing rate was 88.3 ± 16.0%. Angiogenesis and granulation tissue regeneration were increased.	[25]
Chitosan 20 mg/PDA 4.5 mg	15	The wound closure rate was 100%.	[85]
Bacterial cellulose/PDA/polyacrylamide hydrogels	15	Granulation tissue deposition was dense and collagen bundles were regular.	[73]
Dibenzaldehyde-grafted poly(ethylene glycol) (PEGDA), lauric acid-terminated chitosan (Chi-LA), and curcumin (Cur)-loaded mesoporous PDA nanoparticles (PDA@Cur)	14	Most of the wound healed.	[68]
Agarose-PDA hydrogel (APG)	14	The collagen density increased and most of the wound healed.	[117]
MOF-PDA	12	The wound healed.	[74]
Educed PDA nanoparticles (rPDA NPs) incorporated oxidized dextran/chitosan hybrid hydrogels	15	The wound healed with scar tissue.	[118]
pDA/PLGA nanofibrous/platelet-derived growth factor-bb	7	The wound was reduced by >80%.	[119]
Carpacara methacrylate-ZnO/PDA	14	There was complete epithelialization.	[81]
Ordinary medical gauze sequentially with PDA, perfluorocarbon, and silver nanoparticle	14	The wound area was reduced to 8.1 ± 5.7%.	[64]
QCS/reduction graphene oxide-PDA/poly(*N*-isopropylacrylamide)	14	The wound healed completely with re-epithelialization and no scar tissue was visible.	[90]
2D PDA nanosheets	14	The wound of the high-dose group was healed without obvious scarring.	[120]
PDA coated BC with in situ silver nanoparticle reduction	25	The third degree burn wound healed completely without scarring.	[121]
Gelatin/dopamine cryogels	14	The wound was completely healed and re-epithelialized without scarring.	[75]
Bromelain immobilized electrospun poly(ε-caprolactone) (PCL) fibers (BrPDA-PCL fibers)	11	The wound was completely closed with scarring.	[122]
PTA/PDA	18	The wound was completely closed in the full-thickness skin defect model.	[123]
PDA-RGD peptide-bFGF	60	The rabbit ear wound was completely healed and epithelialized without scarring.	[124]
Basic fibroblast growth factor (bFGF)/PDA/poly(lactide-co-glycolide) (PLGA) fibers	14	The wound healing rate was 92% and scar tissue was obvious.	[125]
Zein/PDA/TiO_2_	15	There was complete re-epithelialization with partial scar tissue.	[114]
Van-gel-PDA	46	The burn wound was closed without re-epithelialization.	[126]
Eggshell membrane/PDA	7	The wound healing rate was 81.9%.	[127]
MoS2@PDA-Ag	8	Most of the wound healed.	[55]
EGF-loaded PDA-NP-CS/SF cryogel	21	The wound healed completely and was re-epithelialized.	[128]
H_2_O_2_/HPR (horseradish peroxidase)-PDA-rGo	14	The hydrogel group had relatively more skin appendages and blood vessels such as hair follicles.	[129]
Carbon quantum dot (CQD)-decorated ZnO (C/ZnO) composites were chosen as the functional NPs	10	The skin was intact and the subcutaneous tissue structure was normal.	[77]
Cotton gauge (CG)-coated with quercetin and silver	12	On day 12, a thin layer of dermis was observed complete with glands and hair roots forming as per normal tissue. Connective tissue deposition and adipose tissue formation were enhanced.	[130]
GT-DA/CS/CNT gelatin-grafted-dopamine (GT-DA) and PDA-coated carbon nanotubes (CNT-PDA)	14	The wound surface was almost completely closed and smooth new epidermal tissue appeared.	[78]
